# Real-time clinician text feeds from electronic health records

**DOI:** 10.1038/s41746-021-00406-7

**Published:** 2021-02-24

**Authors:** James T. H. Teo, Vlad Dinu, William Bernal, Phil Davidson, Vitaliy Oliynyk, Cormac Breen, Richard D. Barker, Richard J. B. Dobson

**Affiliations:** 1 Kings College Hospital NHS Foundation Trust, London, United Kingdom; 2 Guys & St Thomas Hospital NHS Foundation Trust, London, United Kingdom; 3 Institute of Psychiatry, Psychology and Neuroscience, Kings College London, London, United Kingdom

**Keywords:** Health care, Public health

## Abstract

Analyses of search engine and social media feeds have been attempted for infectious disease outbreaks, but have been found to be susceptible to artefactual distortions from health scares or keyword spamming in social media or the public internet. We describe an approach using real-time aggregation of keywords and phrases of freetext from real-time clinician-generated documentation in electronic health records to produce a customisable real-time viral pneumonia signal providing up to 4 days warning for secondary care capacity planning. This low-cost approach is open-source, is locally customisable, is not dependent on any specific electronic health record system and can provide an ensemble of signals if deployed at multiple organisational scales.

Analyses of search engine and social media feeds have been attempted for infectious disease outbreaks^1^, but have been susceptible to artefactual distortions from health scares or keyword spamming in social media or the public internet^2–4^. In contrast to the unfiltered unmoderated freetext of the public internet, freetext in electronic health records (EHR) are less tempestuous because of access management to healthcare-professionals-only; text in an EHR is therefore richer in saliency, lower in non-specific noise and less prone to distortions. However EHR’s often have inconsistent data standardisation with structured data mixed with unstructured free text, as well as a hybrid of modern and legacy closed systems. More complex systems using machine learning of observations from structured data have promising accuracy^5^ but would be heavily dependent on hospitals having highly structured and interoperable EHR’s. Traditional data aggregation methods rely on gold-standard cases generated from reporting mechanisms like structured case report forms for local or national registries.

We describe an approach using real-time aggregation of keywords and key phrases from freetext in electronic health records to produce a real-time signal during the Covid pandemic. This open-source system takes text from structured and unstructured fields in near-real-time from clinician-generated documentation in the electronic health records and does not require health data to be standardised into any ontology. This real-time symptom-based unstructured text aggregation could provide earlier warning as it avoids laboratory processing delays and undersampling bias (significant confounders during the early pandemic period).

A query was defined producing an aggregated count of patient documents containing symptom keywords and phrases for symptomatic Covid: (“dry cough”, “pyrexia”, “fever”, “dyspnoea”, “anosmia”, “pneumonia”, “LRTI”, “lung consolidation”, “pleuritic pain” and associated synonyms, acronyms and poecilonyms) normalised against patient documents not containing these phrases or containing negations of the phrases (e.g. “no dry cough”, “no anosmia”). This was used to generate an index of signal enriched for symptom clusters of symptomatic Covid which compares favourably to the gold-standard data of laboratory samples at King’s College Hospital (KCH) and Guys and St Thomas’ Hospital (GSTT) (Fig. 1).

**Fig. 1:**
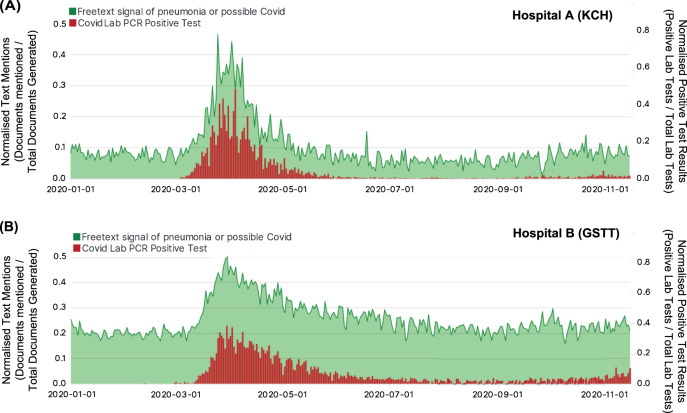
Timeline of freetext signal for Covid-like symptoms from clinician text entries for two major London hospitals during the Covid pandemic. **A** Hospital A (Kings College Hospital, KCH) freetext signal (Green) and positive laboratory tests normalised against negative tests (Red) from 1st January 2020 till 12 November 2020; **B** Hospital B (Guys & St Thomas’ Hospital, GSTT) freetext signal (Green) and positive laboratory tests normalised against negative tests (Red) from 1st January 2020 till 12 November 2020. KCH text signal was derived from both A&E and inpatients, while GSTT text signal was derived only from inpatients (A&E data not accessible). A figure with extension of the time period into January 2021 is available in Supplementary Fig. 6.

Cross-correlation with laboratory samples from each hospital peaked at 4 days at KCH (cross-correlation *r* = 0.783) and GSTT at 0 days before (cross-correlation *r* = 0.757) (Supplementary Fig. 1A) with a good cross-correlation between sites (Supplementary Fig. 1B).

Signal density distribution was also performed to quantify the power of the signal at KCH and GSTT (Supplementary Fig. 2A). As the KCH signal was derived from a mix of sources (A&E admissions and Inpatient text), this was decomposed into two distinct signals as well (Supplementary Fig. 2B) with a higher signal strength in records which have heading prompts to the text boxes (e.g. history, investigations, management) at GSTT Inpatients and KCH A&E compared to records with complete unprompted free text (KCH Inpatients). The decomposed KCH signals had multiple sub-peaks (likely related to weekday–weekend cycles in A&E) and an accumulating correlation for inpatients (due to the persistence of affected patients in hospital) (Supplementary Fig. 2C). This indicates that analogous freetext signals between institutions should not be directly compared. The text signal was also strongly correlated with data on Covid cases using regional datasets for London hospital admissions (Supplementary Fig. 3) while capturing the effects of multiple waves. Of note, seasonal influenza epidemics over the winters from 2018–2020 were detectable due to many overlapping text features (Supplementary Fig. 4) indicating non-specificity for viral pneumonias.

Previous attempts at text-based epidemic forecasting have been largely focused on search engine or social media trends from the public internet to forecast influenza, and to measure such trends against structured public health databases at national level^6–10^. Other approaches for population-level forecasting have used structured manual submissions to national public health systems like the Centre for Disease Control’s Influenza-Like Illness Reports^9^. Our approach does not attempt to forecast but acts as a noisy real-time barometer of local clinical data which is adequate for local operational use at low-cost.

While we show how this approach operates at the scale of single healthcare organisations, it can be scaled to a whole system level either by combining locally generated customised real-time signals from multiple organisations or by centralising the clinical data first before generating the real-time freetext signal from a ‘bag of words’. However depending on the coverage of the local EHR over local care pathways, signals between hospitals may not be directly comparable. As such, we believe an ensemble of locally generated signals is superior as a single organisation is able to customise the locally derived signal for local operational purposes while simultaneously a wider health economy may derive utility from the ensemble of signals for other central planning purposes (mindful of the limits of comparing different organisations).

It is important to note limitations to our approach – firstly, ecological real-time freetext data is uncurated, and could be susceptible to distortions as certain phrases in freetext could produce artificial distortions (e.g. if ward names, job titles or operational pathways are named “Covid”). This risk is minimised in this study as the word “Covid” was not included from the signal in this study, but this could be tailored to the local context or dialect.

Secondly, one can only aggregate and harness knowledge that clinicians and patients consider of sufficient relevance or saliency to record; loss of smell and anosmia has been described in Covid^11^. Our text aggregator was able to detect an initial attenuated signal in the freetext in March 2020 without the benefit of recall bias (Supplementary Fig. 5). The converse is true, the signal was subsequently amplified by the media as subsequent scientific publication of the findings of anosmia on 17/05/2020 caused a second surge in the incidence of these phrases likely due to increasing clinician awareness.

This media-sensitive signal highlights that incident clinical language could become self-fulfilling–heightened awareness may increase the use of such words even in clinical text either through speculative differentials or checklists. We managed this by eliminating negation terms, combining multiple symptom phrases and focusing on formal documentation only (e.g. clinical episode summaries). Nonetheless an organisation should exercise caution against disseminating the exact words or phrases used to generate the signal to minimise ‘hashtag meme’-like or ‘trending’ phenomenon amongst clinicians. Additionally, this means that the freetext signal would only be useful for evaluating prevalence during periods where there is no time-varying potentiation on clinician record-keeping by media or local policies.

Thirdly, this study as proof-of-concept was confined to secondary care settings; during the Covid first wave in March 2020, there were triage mechanisms to redirect low severity cases away from secondary care affecting visibility. For a global view of a health economy, text aggregators would also need to be deployed in primary care and emergency care providers.

In summary, we report a natural language approach of real-time clinical data that is flexible and scalable to feed dashboards of activity for capacity planning and can be generalised across organisations to provide early warning of future pandemic surges. This low-cost approach is open-source, is not fixed into any specific EHR system. It might be possible to deploy this at multiple organisations to provide an ensemble of signals.

## Methods

### Platform

The text feed was generated by a locally deployed Cogstack instance^12^ at Kings’ College Hospital NHS Foundation Trust, UK consisting of an open-source toolkit for extracting text from EHR’s (https://github.com/CogStack). A subsequent instance was deployed at Guys & St Thomas’ Hospital NHS Foundation Trust, UK as validation in another hospital with different care pathways, different EHR and sociodemographics.

### Text feed

A query was defined producing an aggregated count of patient documents containing symptom keywords and phrases for symptomatic Covid: (“dry cough”, “pyrexia”, “fever”, “dyspnoea”, “anosmia”, “pneumonia”, “LRTI”, “lung consolidation”, “pleuritic pain” and associated synonyms, acronyms and poecilonyms) normalised against patient documents not containing these phrases or containing negations of these phrases (e.g. “no dry cough”). This produces a signal which is enriched for symptom clusters of symptomatic Covid. Of note, affirmative diagnostic phrases (e.g. “confirmed Covid”) were excluded from the query in this manuscript although these are used in practice to boost the signal. No machine learning was used to derive the query.

Presence of positive COVID PCR laboratory samples was used as the gold standard signal of cases. A similar query was constructed with local adaptations within a parallel Cogstack instance at GSTT.

### Legal and ethical use of patient data

This project was conducted under public health requirements and operational service delivery of the hospitals as part of the UK’s NHS (Control of Patient Information Regulations) 2002 (COPI) regulation to share confidential patient information to process under for COVID-19 purposes. Identifiable data was not evaluated. Additional guidance and support was sought from the patient-led Kings Electronic Records Research Committee (KERRI) which operates under an opt-out model under London South East Research Ethics Committee approval (reference 18/LO/2048).

### Reporting summary

Further information on research design is available in the Nature Research Reporting Summary linked to this article.

## Supplementary information

Supplementary Information

Reporting Summary

## Data Availability

Only aggregated counts over time is available (no patient-level data due to privacy). Any request for this will need to be reviewed first by the hospital information governance committee which includes the Caldicott Guardian and Data Protection Officer.

## Code availability

Source code for the platform is available at (https://github.com/CogStack).
