# Supplementary material for: Real-time clinician text feeds from electronic health records
Source: NPJ Digit Med. 2021 Feb 24;4:35. doi: 10.1038/s41746-021-00406-7 (PMC7904856; doi:10.1038/s41746-021-00406-7)

Supplementary Figure 1A: Cross-correlation of GSTT freetext signal with lab positive samples, peak 0 day lag of ~0.757, and KCH combined signal with KCH lab positive samples, peak 4 day lag of ~0.783.

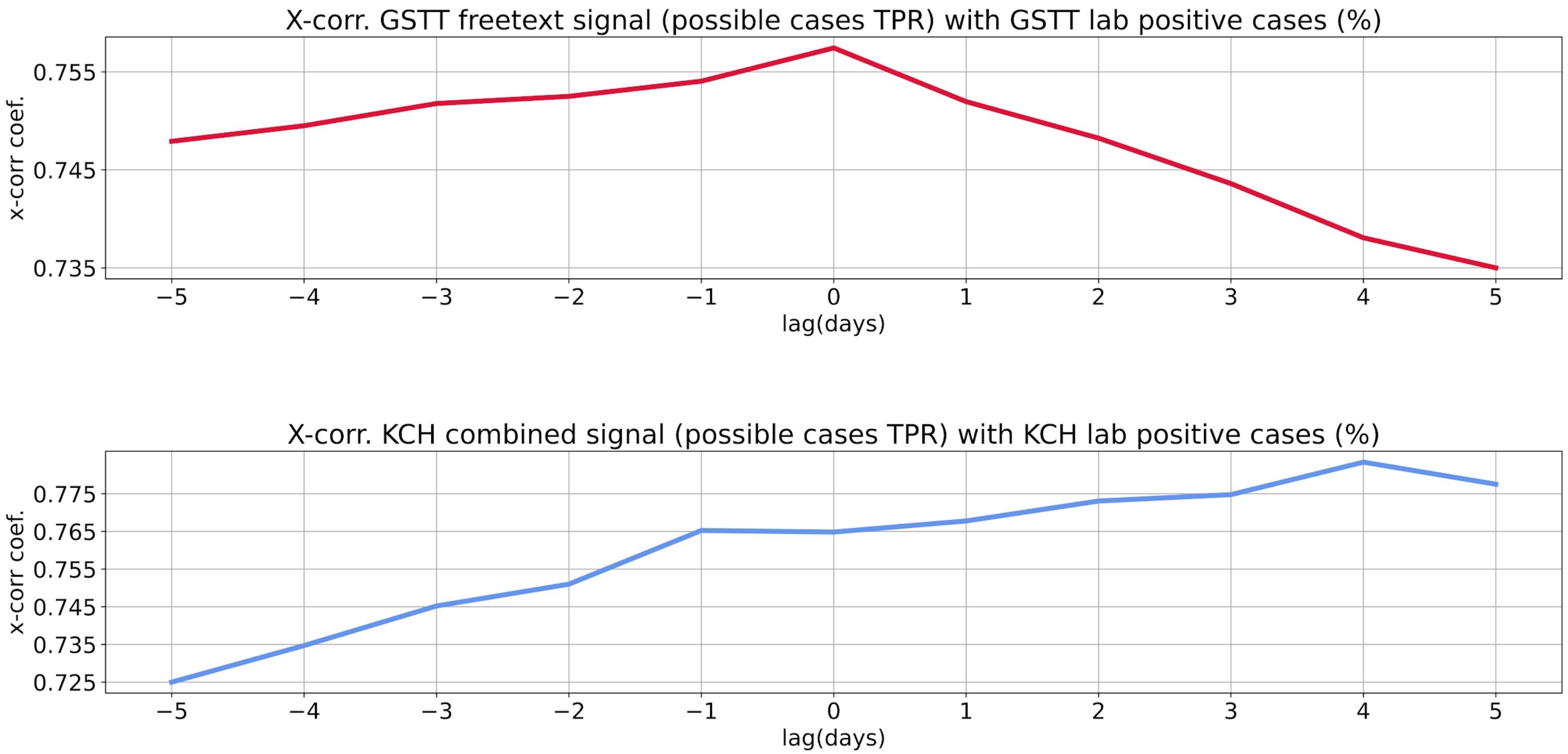

Supplementary Figure 1B: Cross-correlation of GSTT freetext signal with KCH freetext signal.

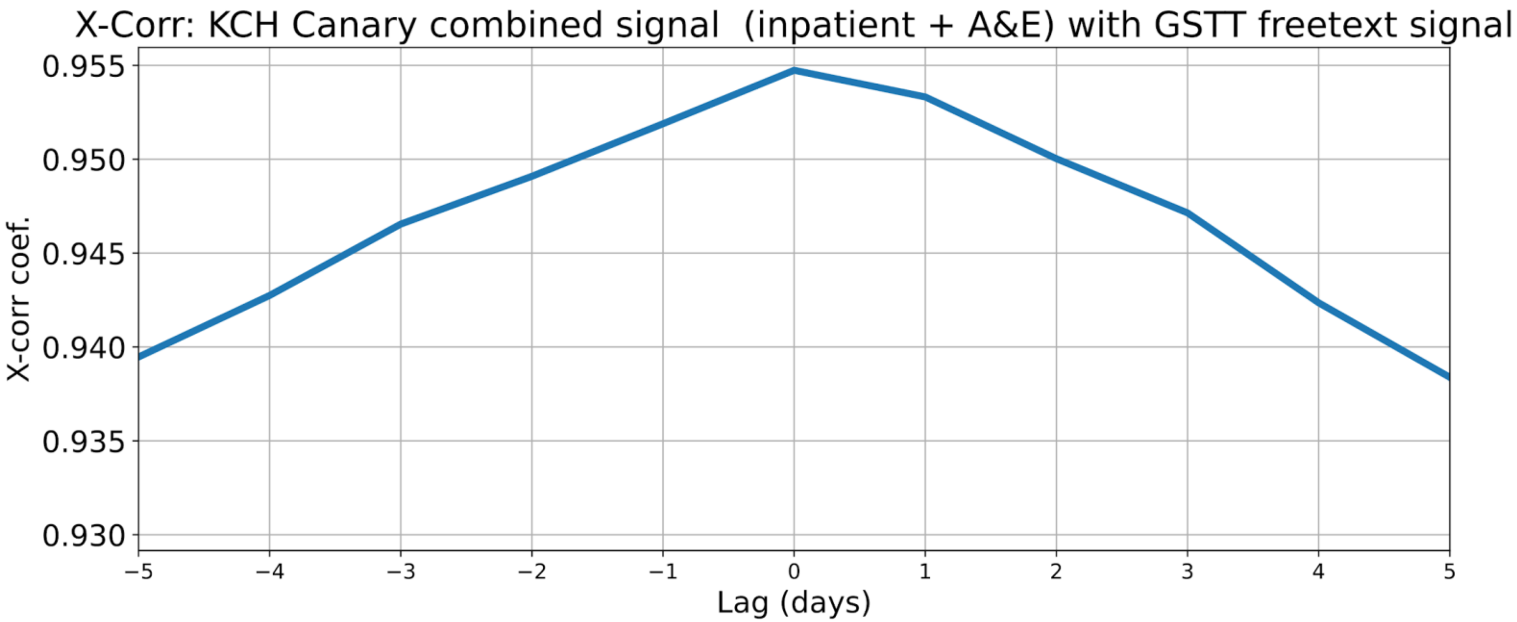

Supplementary Figure 2A: Probability density distribution using the Kernel Density Estimation for both KCH and GSTT freetext signals up till 12/11/2020 indicating substantial signal strength coinciding with the first curve.

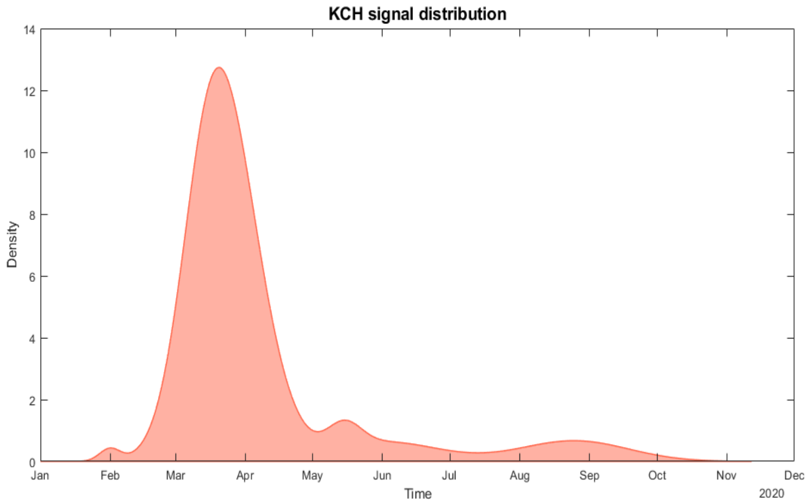

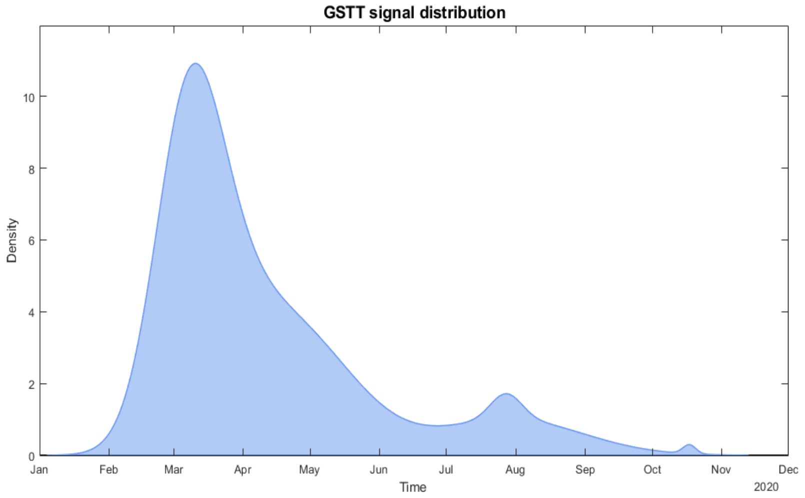

Supplementary Figure 2B: Comparing the combined strength of the KCH freetext signal (A&E + Inpatient) to freetext GSTT (Inpatient only). In GSTT and KCH A&E, the free text was from text boxes with heading prompts for history, examination findings, investigations, management plan and such. In KCH Inpatient, the text from freeform text inputted into text boxes without any heading prompts. Text from GSTT A&E records was not available for this work.

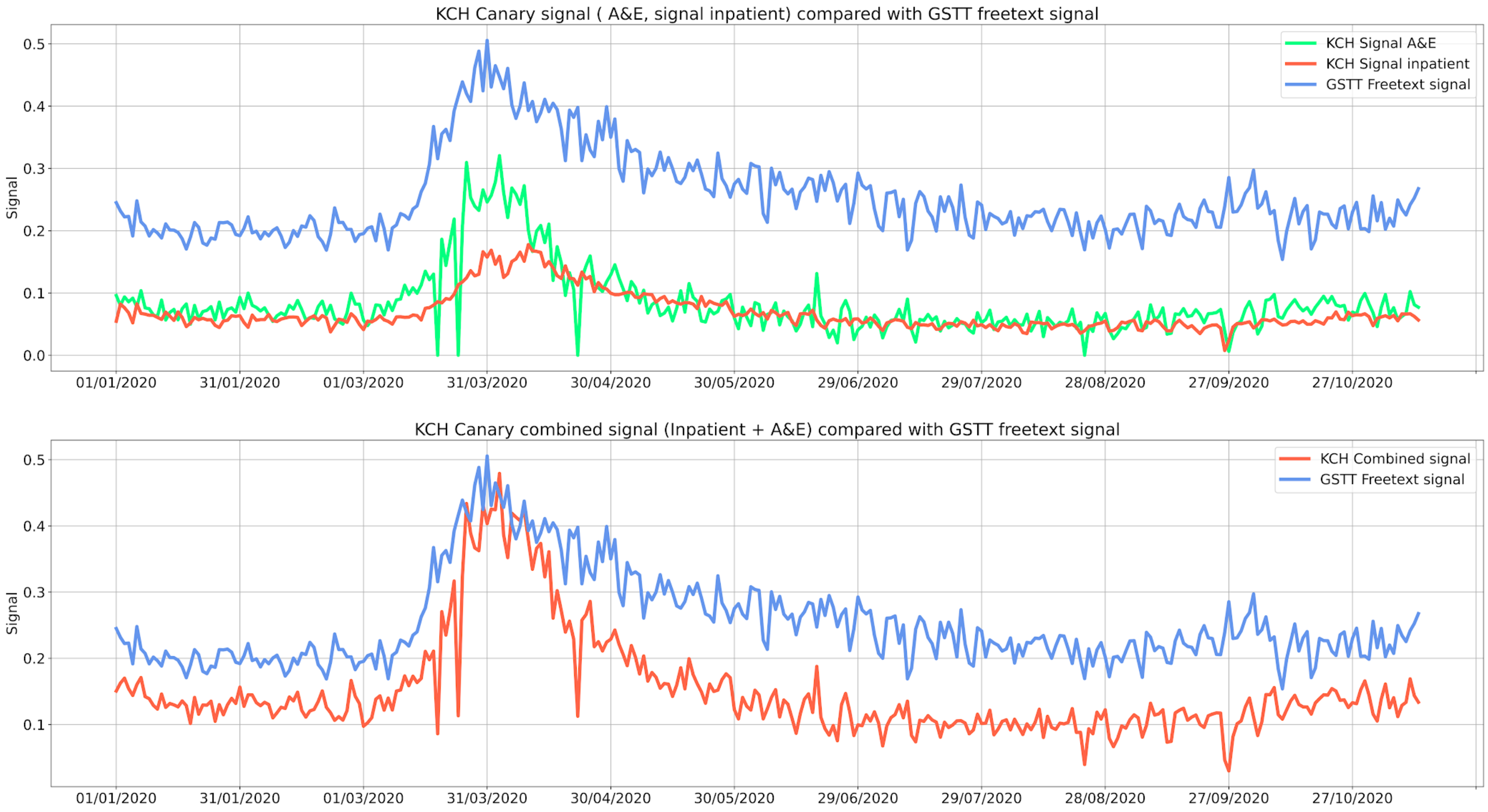

Supplementary Figure 2C: Cross-correlation of KCH freetext signal decomposed into two signals: signal from A&E only (top, red, cross-correlation peak at 4 days at 0.798) and freetext signal from inpatient only with lab positive samples (bottom, blue, cross-correlation peak at 4 day lag at

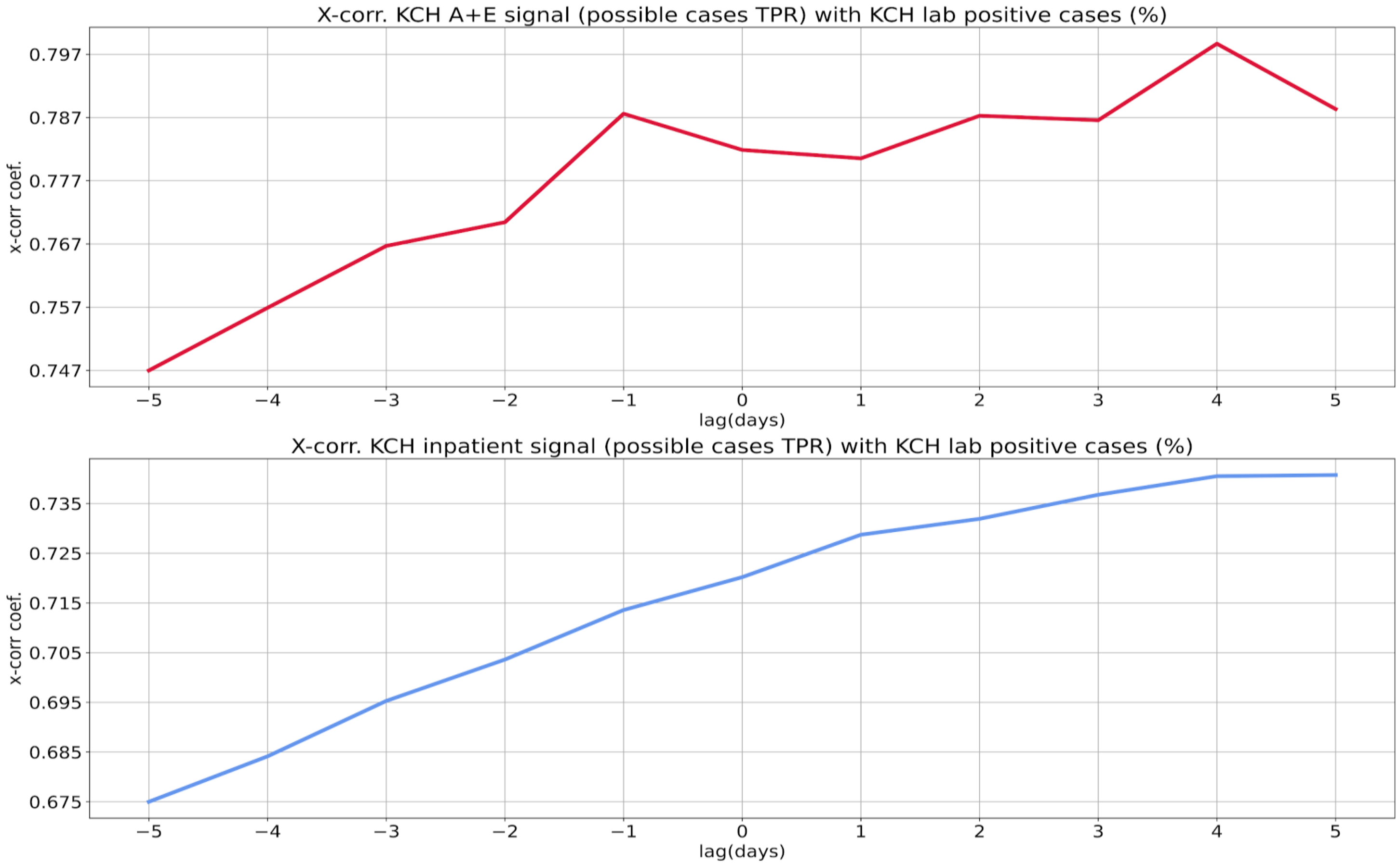

0.740).

Supplementary Figure 3: Scatterplot of the freetext signal from King's College Hospital with the London regional hospital admissions from 19/03/2020 (first date of the datastore) till 12/11/2020 showing a strong positive correlation (Pearson correlation, r=0.912) between the signal in a single hospital with a region-wide hospital admissions

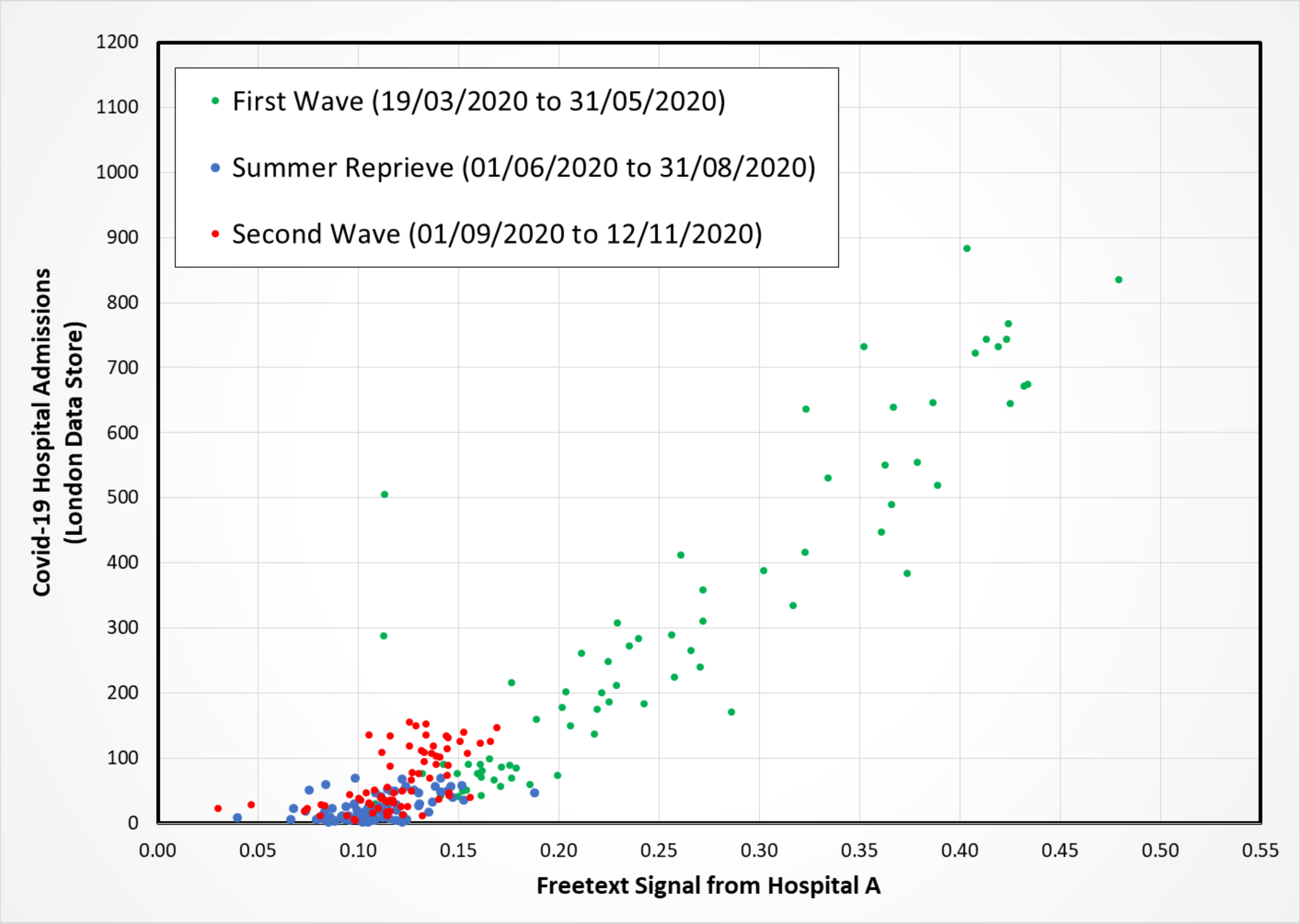

Supplementary Figure 4: Real-time aggregation of clinical text feeds showing that the seasonal influenza symptoms are also being detected by viral pneumonia symptom aggregators (bottom) coinciding with influenza laboratory testing (top); cross-correlation for 01/01/2017 till 29/02/2020, r = 0.414 for 0 day lag, r = 0.420 for -1 day lag, r=0.348 for -2 day lag).

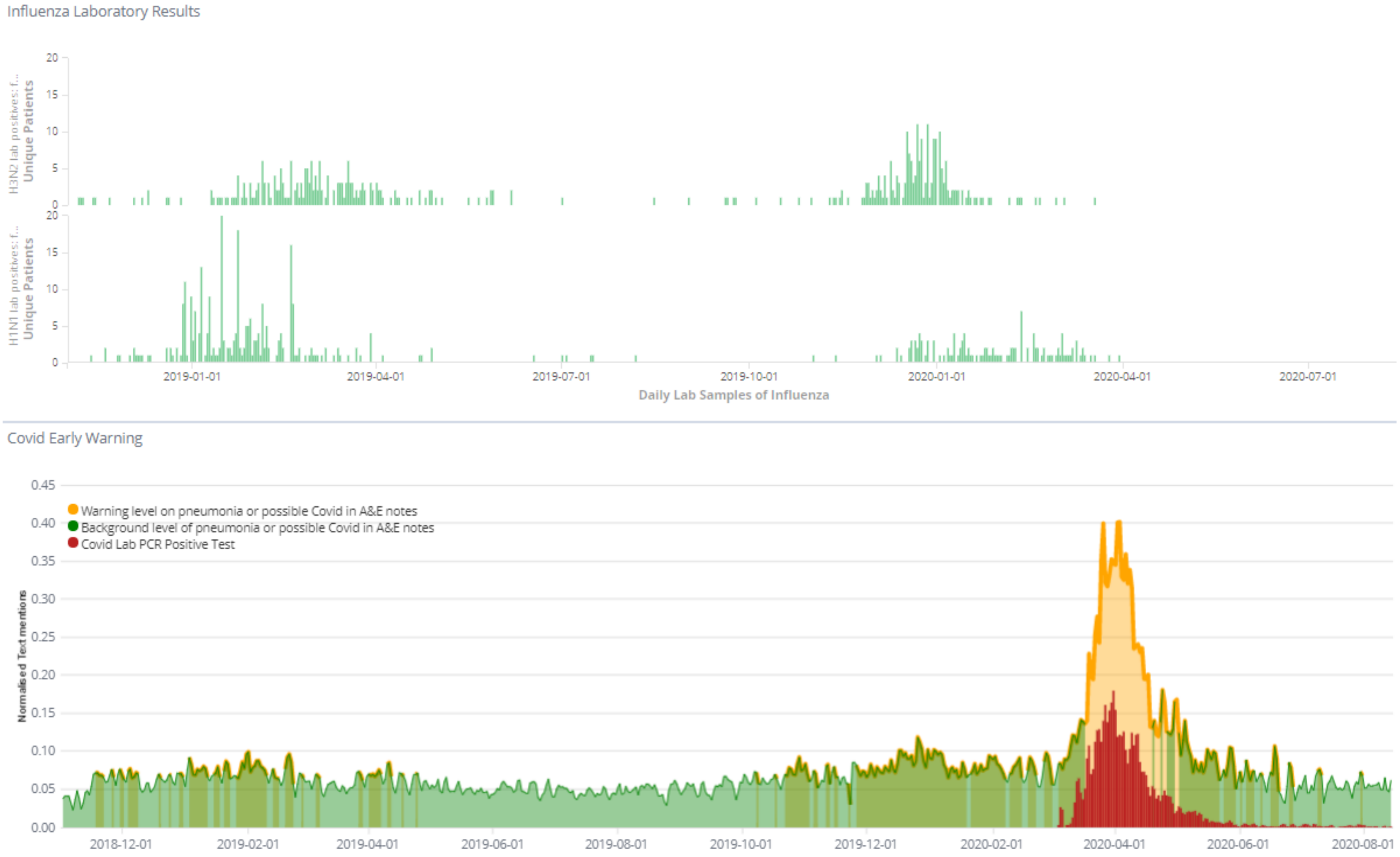

Supplementary Figure 5: Real-time aggregation of key phrases “Anosmia”, “Loss of Taste” and “Loss of Smell” with simple negations only (e.g. “No anosmia”). Note that the detection of these terms started in March 2020 during the first Covid surge consistent with the proportion of cases, followed by a second upswell from 17/05/2020 (vertical red line) coinciding with the publication of Nature Medicine article confirming association through an app-based study [10].

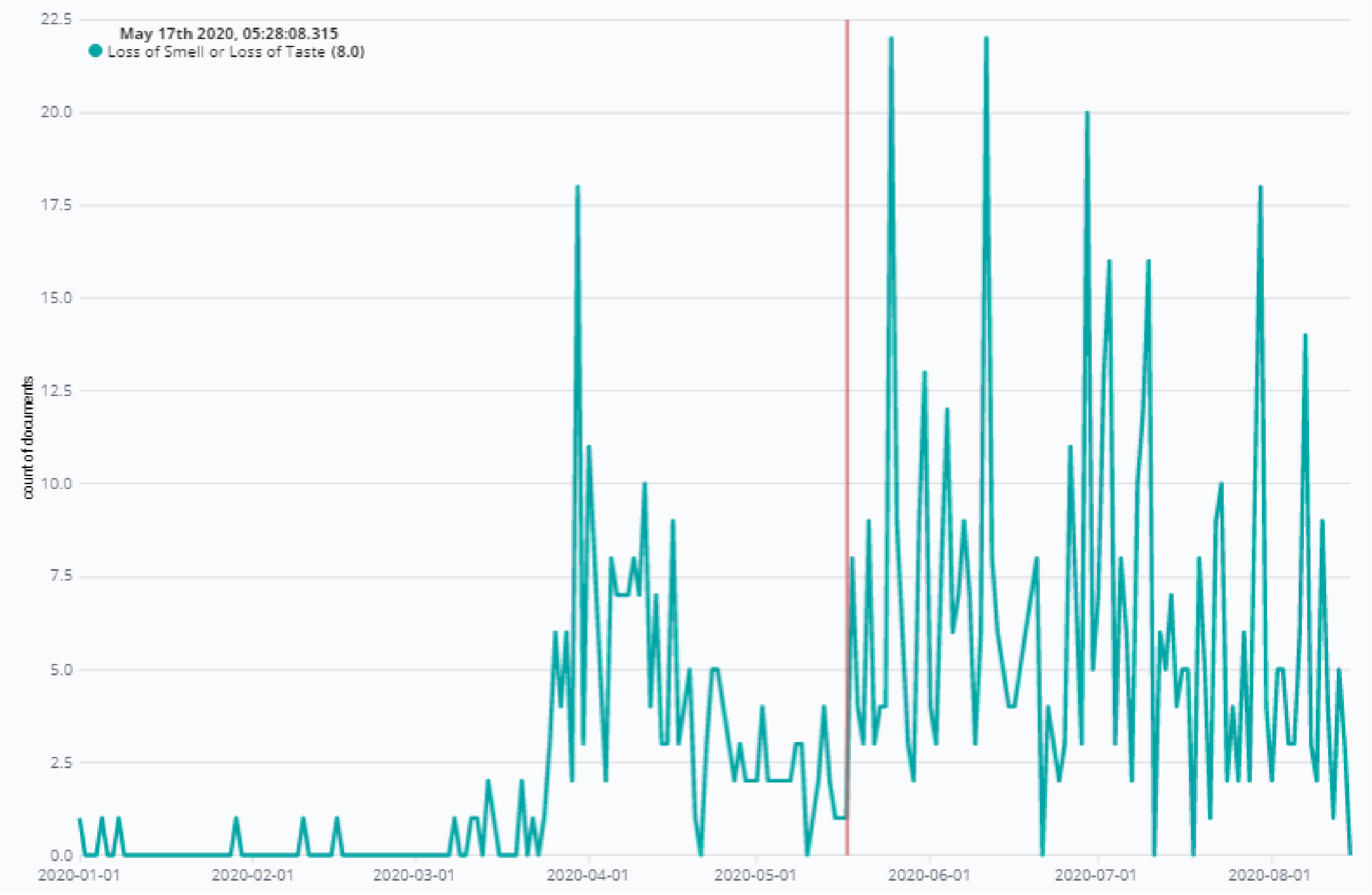

Supplementary Figure 6: Updated figu res as of 19/01/2021 to include most of the London second wave at KCH (top) and GSTT (bottom), with freetext signal (green) scaled on the left y-axis and lab test result (red) scaled on the right y-axis

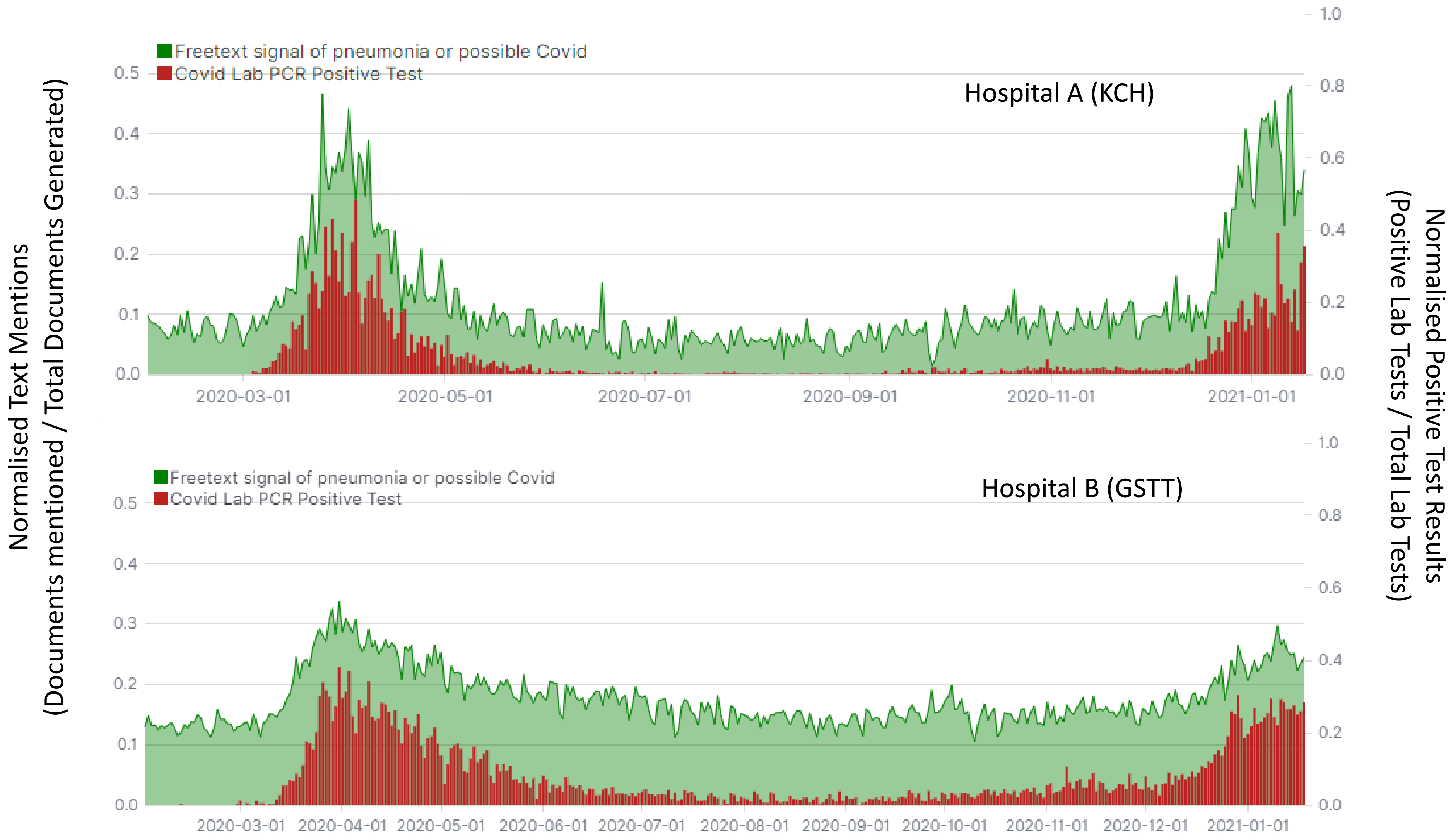

Supplement: Supplementary file 1 — Supplementary Information [file 41746_2021_406_MOESM1_ESM.pdf]
